# Wide-temperature rechargeable Li metal batteries enabled by an in-situ fabricated composite gel electrolyte with a hierarchical structure

**DOI:** 10.1016/j.fmre.2021.10.003

**Published:** 2021-11-03

**Authors:** Chao Ma, Xizheng Liu, Hui Geng, Xiaoshu Qu, Wei Lv, Yi Ding

**Affiliations:** a Tianjin Key Laboratory of Advanced Functional Porous Materials, Institute for New Energy Materials and Low-Carbon Technologies, School of Materials Science and Engineering, Tianjin University of Technology, Tianjin 300384, China; b Shenzhen Geim Graphene Center, Engineering Laboratory for Functionalized Carbon Materials, Tsinghua Shenzhen International Graduate School, Tsinghua University, Shenzhen 518055, China; c Jilin Institute of Chemical Technology, Jilin 132073, China

**Keywords:** Lithium metal anode, Hierarchical gel electrolyte, In-situ polymerization, Wide temperature, LiGaIn alloy

## Abstract

Lithium metal batteries (LMBs) are well recognized as promising next-generation high energy density batteries, but the uncontrollable Li dendrites growth and the volatilization/gas production of electrolytes, which become extremely worse at low and high temperatures, restrict their practical utilizations. In this work, a hierarchically structured polymerized gel electrolyte (HGE), which was composed of an inorganic (Li_x_Ga_86_In_14_ alloy and LiCl salt)/organic (polymerized tetrahydrofuran (THF)) hybrid layer and the bulk polymerized THF electrolyte, was proposed to achieve a steady performance of LMBs over a wide temperature range of -20-55 °C. The HGE fabrication can be completed within assembled cells with a simultaneously occurring replacement-polymerization-alloying reaction, which helps decrease the interfacial resistance and enhance the stability and ion diffusion under both low and high temperatures. The use of THF with low polarity also ensures high ion conductivity under low temperatures. With such HGE, the Li symmetric cells showed low overpotential under 10 mA/cm^2^ with a capacity of 10 mAh/cm^2^ over a 1200 h cycling, and the full cell coupled with Li_4_Ti_5_O_12_ demonstrated high capacity retention over 5000 cycles at room temperature. Besides, the symmetric cells showed low overpotentials of 12 mV at 55 °C and 80 mV at -20 °C at 2 mA/cm^2^ after a 1000 h cycling, and the full cell revealed the high capacity retention of 93.5% at 55 °C and 88.8% at -20 °C after 1500 cycles under a high current density of 1000 mA/g. This work shows a hierarchically structured polymerized electrolyte design for advanced Li batteries workable under broad temperatures.

## 1 Introduction

The rapid development of electric vehicles and smart grids requires batteries with high energy density, safety and ability to work over a wide temperature range [1], [2], [3]. Lithium metal batteries (LMBs) that use Li metal anode with high specific capacity are the most promising systems to satisfy the above-increasing demands [4, 5]. However, the safety of LMBs is hard to meet the requirements for practical uses since the Li dendrites formed by the heterogeneous Li deposition can pierce the separator and lead to the short circuit [6], [7], [8], [9], [10], [11]. Extensive efforts have been carried out to solve the above problems, such as fabricating three-dimensional (3D) current collectors to decrease the local current density and alleviate the volume expansion [12], [13], [14], [15]. manufacturing a robust protective layer to suppress side reactions with electrolytes [16], [17], [18], and constructing a lithiophilic alloy layer to promote fast and uniform Li deposition [[19], [20], [21], [22], [23], [24], [25]]. However, an urgent concern is that the reversible and stable Li stripping/deposition becomes more complicated under low or high operating temperatures. When the batteries are subjected to lower operation temperatures, the increased electrode polarization and ionic desolvation energy lead to severe dendrite growth [26, 27]. With a high temperature, undesirable side reactions between the Li anode and electrolyte will be further catalyzed by the freshly exposed Li metal, which results in severe accumulation of “dead Li” and fast electrolyte decomposition [28, 29]. Thus, the usability of the above methods to suppress dendrite growth becomes uncertain under wide-temperature working conditions.

Using gel-like polymer electrolytes to replace liquid electrolytes (LEs) is an effective solution to enhance the thermal stability and keep a stable electrolyte/electrode interface, which can ensure the cycling stability of LMBs at a high temperature [32], [33], [34]. However, the severe ionic conductivity decrease at a low temperature of these electrolytes significantly deteriorates the battery performance. The polymerization of electrolytes with lower polarity should be a potential method to balance the electrochemical performance of gel-like polymer electrolytes under high and low temperatures as the low polarity solvent can significantly enhance ion diffusion at a low temperature [30, 31]. However, the ion transfer across the gel electrolyte/electrode interface should also be concerned because the solid-solid contact between them leads to large ion diffusion resistance, which becomes even worse under low temperatures due to the solidification of solvents in the gel.

Herein, we design a hierarchically structured polymerized gel electrolyte (HGE), which contains an inorganic (Li_x_Ga_86_In_14_ alloy and LiCl salt) /organic (polymerized THF) hybrid layer and the bulk polymerized THF electrolyte, to solve the above problems. The use of THF with low polarity ensures high ion conductivity even under low temperatures. The polymerized THF in the hybrid layer and bulk is prepared by the in-situ polymerization in the assembled cell, leading to integrated interfaces and decreasing the interfacial resistance. Moreover, the in-situ polymerization also stabilizes the Li_x_Ga_86_In_14_ alloy and LiCl salt in the hybrid layer, which endows their uniform distribution on the LMA surface and helps form a robust interfacial layer, effectively regulating the Li ion diffusion and suppressing the dendrite growth. Thereby, the assembled Li symmetric cells deliver a small overpotential of 100 mV at 10 mA/cm^2^ with a high capacity of 10 mAh/cm^2^, and can be stably cycled for more than 1200 h. Besides, low overpotentials of 12 mV at 55 °C and 80 mV at -20 °C under the current density of 2 mA/cm^2^ are observed after a 1000 h cycling. It should be noted that the assembled cells coupled with Li_4_Ti_5_O_12_ present high capacity retention of 93.5% at 55 °C and 88.8% at -20 °C after 1000 cycles, highlighting the importance of the above HGE in increasing the usability of LMAs over a wide temperature range.

## 2 Experimental section/methods

### 2.1 Preparation of HGE

0.5 M Lithium bis(trifluoromethanesulfonyl)imide salt (LiTFSI, 99%, Innochem) was dissolved in Tetrahydrofuran (THF, 99.9%, extra dry with molecular sieves, Innochem) solution and used as the liquid electrolyte for polymerization unless otherwise specified. Then, 0.086 M Gallium (III) chloride (GaCl_3_, ultra-dry, 99.999%, Alfa) and 0.014M Indium (III) Chloride (InCl_3_, 99.99%, Innochem) were added to the above LiTFSI-THF electrolyte, and after that, 1% volume ratio of 1,2-Epoxypropane (PO, 99%, Adamas) was introduced to promote the polymerization process. Finally, the batteries were assembled with the above electrolyte containing Ga^3+^/In^3+^ and rested for 12 h at room temperature to form HGE.

### 2.2 Battery assembly and test

All the coin cells (CR2032-type) were assembled in an Ar-filled glovebox, using Glass-fiber (GF) as separator. Symmetric cells were assembled with two pristine Li sheets and separated by a GF separator. Cells with pure liquid 0.5 M LiTFSI/THF electrolyte or HGE were assembled for comparison. The pro-type Li|LTO cells consisted of a Li_4_Ti_5_O_12_ electrode, metallic Li, and different electrolytes. The commercial LTO had a mass loading of ∼1.0 mg/cm^2^ on the Al current collector. Galvanostatic discharge–charge tests were performed on the battery testing system (LAND CT2001A) at 20 °C, -20 °C and 55 °C. The cycled cells were disassembled in the glovebox, and the Li metal electrodes were harvested and rinsed with pure THF before analysis.

### 2.3 Material characterizations

X-ray diffraction (XRD, MiniFlex 600, Rigaku) was used to verify the structure and composition using Cu Kα radiation at a scan rate of 5° min^−1^ over a 2θ range of 30°–70°. The HGE and cycled electrodes were covered by Kapton film to prohibit exposure to ambient air during XRD testing. The thermal stability was measured on thermo-gravimetry differential thermal analysis (TG-DTA, 209F3A, NETZSCH) from 40 to 200 °C with a heating rate of 10 °C min^−1^ under N_2_ atmosphere. The morphology was observed using a scanning electron microscope (SEM, Verious 460L, FEI) and transmission electron microscope (TEM) with a probe corrector (Titan Cubed Themis G2 300, FEI). The valence states and surface chemistries were characterized using an X-ray photoelectron spectroscope (XPS, ESCALAB 250Xi, ThermoFisher Scientific) equipped with a source gun type of Al-K Alpha. Ar^+^ sputtering was used at ion acceleration of 1 kV to obtain the depth profiles. Fourier transform infrared (FTIR) and Raman spectra were obtained using Perkin Elmer Frontier Mid-IR and Horiba Evolution, respectively. The nuclear magnetic resonance (NMR) spectroscopy was recorded by Avance III HD 400MHz, Bruker and using Chloroform-d solvents.

## 3 Result and discussion

**Fig. 1:**
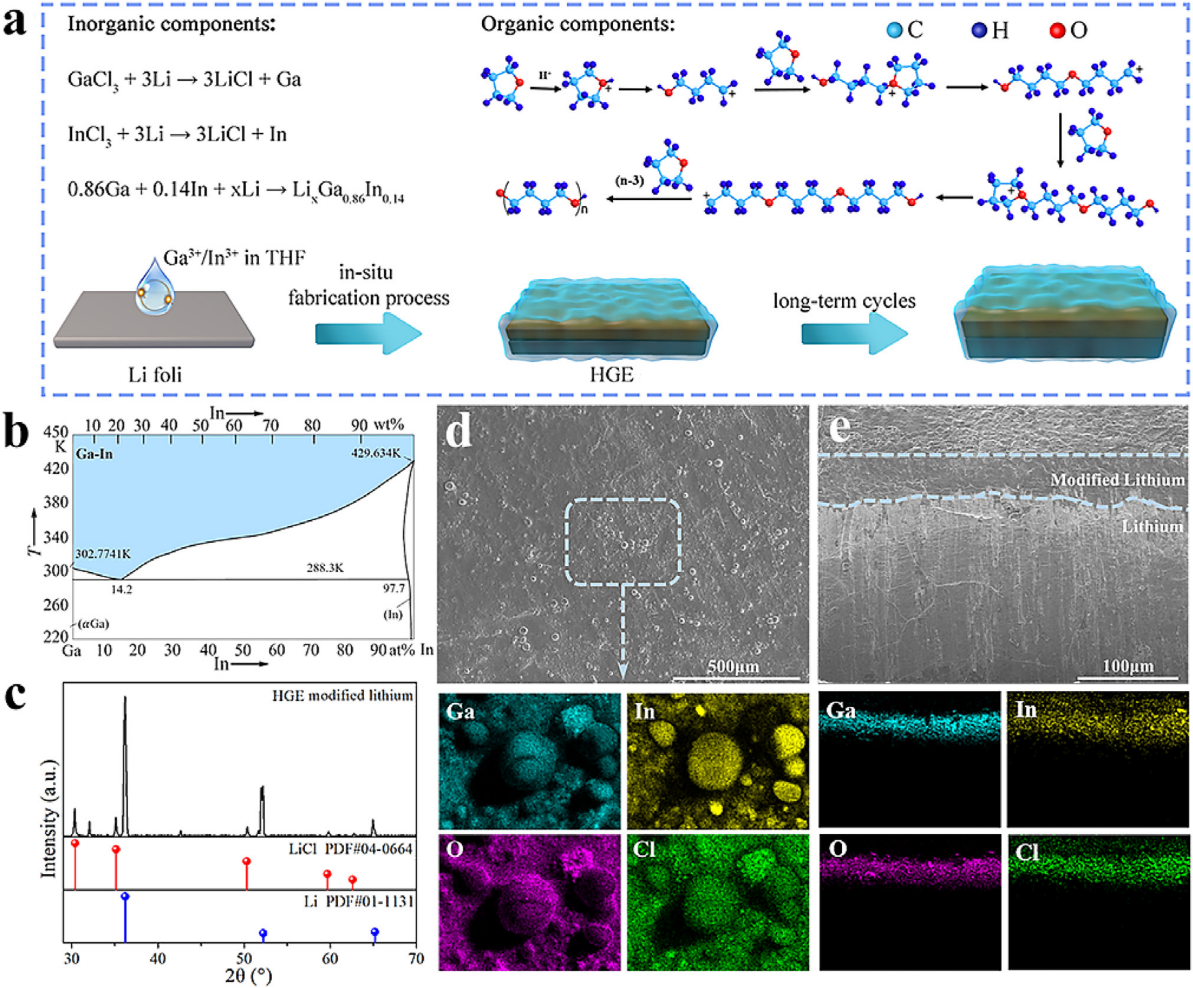
**The preparation and characterization of HGE modified LMAs.** (a) Schematic illustration of the in-situ formation of HGE through a replacement-polymerization-alloying process on LMAs. (b) The phase diagram of Ga-In. (c) X-ray diffraction patterns of HGE modified LMAs. (d-e)SEM images and EDS mappings of Ga, In, O and Cl recorded at the surface (d) and cross-section (e).

The preparation of HGE was achieved by an in-situ polymerization process schematically shown in Fig. 1a, which includes the replacement-polymerization-alloying reactions simultaneously. The Ga^3+^/In^3+^ ions with the fixed ratio dissolved in the THF were reduced by metallic Li and formed a Li_x_Ga_86_In_14_ alloy and LiCl salt on the LMA surface. Note that the ratio between Ga^3+^/In^3+^ was selected according to the phase diagram of Ga-In in Fig. 1b. The Ga_86_In_14_ has a melting point of 288.3 K and thus exhibits a liquid state near room temperature. Meanwhile, the ring-opening polymerization of THF occurred with PO additive. As shown in Fig. S1, PO with higher nucleophilicity would preferentially form coordination intermediates with GaCl_3_. Subsequently, the ring-opening polymerization of THF was triggered by the coordination intermediates formed between GaCl_3_ and PO, as well as LiCl salt. The addition ratio of PO was investigated and 1 vol% was adopted in the following experiments (Fig. S2). The amounts of GaCl_3_ and InCl_3_ were also optimized and the ratio near the GaIn eutectic point was proved to effectively promote the polymerization of THF (Fig. S3). Normally, due to the large surface energy, the formed GaIn alloy cannot uniformly spread on the LAM surface, which can be proved by the SEM shown in Fig. S4. However, in the above process, in situ polymerization of THF helps fix the simultaneously formed Li_x_Ga_86_In_14_ and LiCl salt by the gel network on the LMA surface, realizing their uniform distribution and forming a robust inorganic/organic hybrid layer for homogeneous Li deposition. In traditional batteries, the electrolytes volatilization occurred at high temperatures and thus battery performance deterioration. In comparison, the gel electrolyte can effectively solve this problem, thereby providing enhanced performance over a wide temperature range.

The HGE modified Li anode was characterized by XRD, and the main peaks can be indexed to the metallic Li and LiCl (Fig. 1c). The morphology and elemental distributions were characterized using SEM and energy-dispersive X-ray spectroscopy (EDS), shown in Fig. 1d-e and Fig. S5, respectively. A smooth and dense surface layer with a thickness of tens micrometers can be observed, and Ga, In, and Cl elements are uniformly distributed on the surface. Some GaIn particles can be observed on the top surface because of their large surface energy-induced aggregation. The cross-sectional EDS elemental mappings in Fig. 1e further indicate the uniform distribution of the above elements in the hybrid layer, suggesting the in situ formed THF gel avoids the aggregation of the formed alloys effectively.

**Fig. 2:**
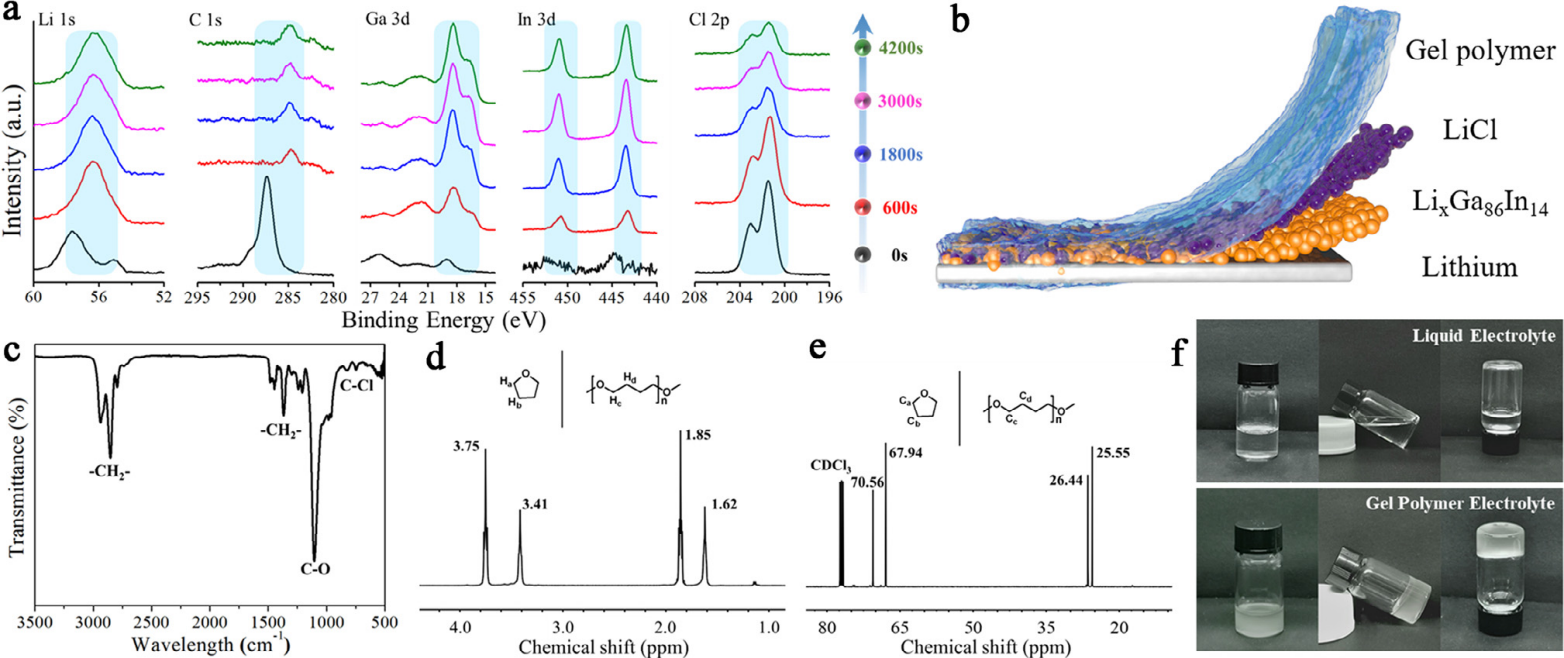
**Physical characterization of HGE.** (a) The XPS depth profiles of Li 1s, C 1s, Ga 3d, In 3d and Cl 2p, respectively. (b) The scheme of possible distribution of each component within the HGE. (c-e) FTIR spectrum, ^13^C and ^1^H NMR spectra of HGE. (f) Optical photographs of electrolytes before and after polymerization.

Systematic investigations were performed to determine the structure and components of the HGE. In-depth XPS profiles shown in Fig. 2a exhibit the elemental distributions of HGE. For the Li 1s spectra, peaks appeared at about 55.1 and 55.7 eV, corresponding to inorganic compounds (such as Li_2_CO_3_ and LiOH) and LiC_x_, which are the reaction products of the HGE with LMAs [35, 36]. After etching the surface layer, the Li 1s peaks shifted to 56.4 eV, which is belonged to the Li 1s peak of LiCl. In the subsequent successive etching process, the peaks remained stable, showing the existence and uniform distribution of LiCl in the HGE. These results were also consistent with the C 1s spectra. For the pristine surface of HGE modified LMAs, the peaks that appeared at about 287.4 and 289.5 eV could be attributed to C-O bond and C-F bond formed by the decomposition of LiTFSI [37, 38]. After etching the surface layer, the binding energy of C 1s peaks shifted to about 284 eV and remained stable, which should be derived from the C-C bond in *n*-[−O−CH_2_−CH_2_−CH_2_−CH_2_−]−*n*. Meanwhile, negligible Ga and In signals were initially detected at the surface, but strong peaks appeared after etching. The maximum intensity of Ga peak was observed after etching for 4200 s, where the maximum intensity for Cl peak was reached after etching 600 s. Based on these results, we can speculate a component distribution in the hybrid layer similar to the structure illustrated in Fig. 2b. The Li_x_Ga_86_In_14_ alloy layer is located close to the LMA surface and gradually becomes less to the top surface due to the decrease of the Li source for the replacement reaction and alloying. The inorganic LiCl-rich layer is mainly found at the middle and the bottom of the hybrid layer, which helps to prevent the reduction of Li^+^ on the top surface due to its electronically insulating property. Thereby, the LiCl and Li_x_Ga_86_In_14_ alloy fabricated a gradient distribution across the HGE, and their synergy effect migrated the Li ion diffusion through the hybrid layer, resulting in a uniform and compact Li deposition. The composition of the formed gel electrolyte was studied by using FTIR as shown in Fig. 2c. The peaks associated with the stretching vibrations of –CH_2_-, C–O, and C–Cl are found at 2937/2854/1445, 1107, and 825 cm^−1^, respectively, which are consistent with the reported polymerized THF [39]. The Raman spectra in Fig. S6 are also consistent with the FTIR results and further substantiate the formation of gel electrolytes. Additionally, the ^1^H and ^13^C NMR were performed to demonstrate the evolution of the chemical environment before and after polymerization (Figs. 2d, e**)** and S7) [40]. The ^13^C and ^1^H spectra confirm that the HGE are polymerized from THF and have a molecular structure of *n*-[−O−CH_2_−CH_2_−CH_2_−CH_2_−]−*n*. Additionally, the NMR results also exhibit the peaks of free THF, suggesting a small amount of THF solvent still exists in HGE, which can further enhance the ion diffusion in the gel electrolyte. The phase transition behavior for HGE was characterized by differential scanning calorimetry (DSC) analysis, as shown in Fig. S8. A typical peak associated with the glass transition temperature (T_g_) is observed at ∼ -30 °C. The crystallization is an important determinant of electrolytes, and low T_g_ of HGE ensures high ionic conductivity at low temperatures. Digital photos of HGE at different temperatures further visualized this phenomenon. As shown in Fig. S9, the prepared HGE was stable between -40 °C and 80 °C and only a slight phase transition occurred above 80 °C, which confirms the environmental stability of HGE and thereby guarantees the wide-temperature electrochemical performance. Fig. S10 depicts the thermal stability of the prepared HGE. It is found that the mass loss mainly occurred at 150 °C, indicating the improved thermal stability compared with its liquid counterpart and the usability of HGE at high temperatures.

**Fig. 3:**
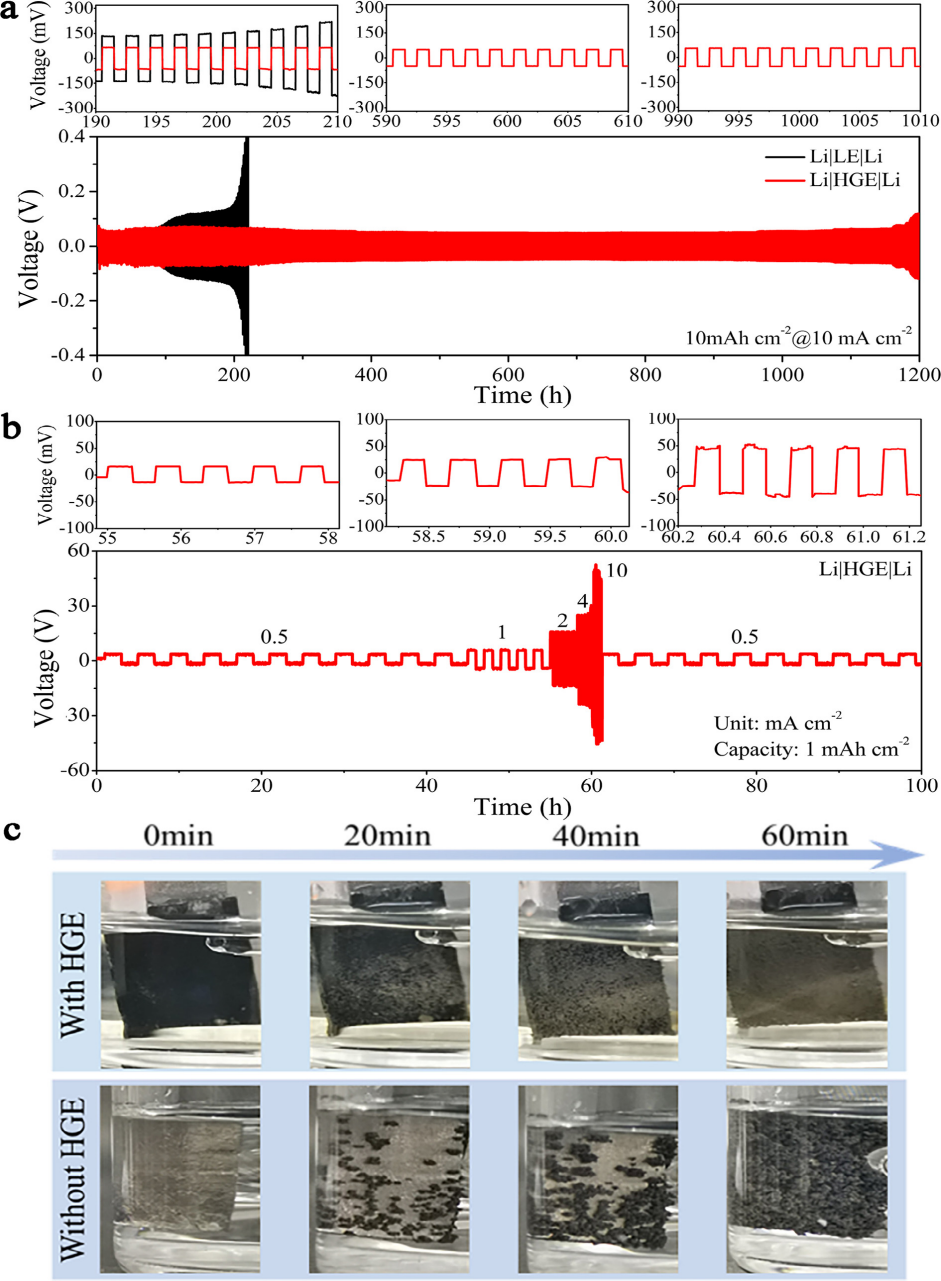
**Electrochemical performance of HGE modified LMAs in symmetric cells and digital photos of discharged LMAs with/without HGE protection.** (a) Galvanostatic cycling performance with 10 mAh/cm^2^ at 10 mA/cm^2^ and its selected stripping/plating curves. (b) Rate capabilities at current densities of 0.5, 1, 2, 4, 10, and 0.5 mA/cm^2^ with a limited capacity of 1 mAh/cm^2^ and corresponding selected curves. (c) Digital photos with different plating times of bare and HGE modified LMAs at a current density of 5 mA/cm^2^.

**Fig. 4:**
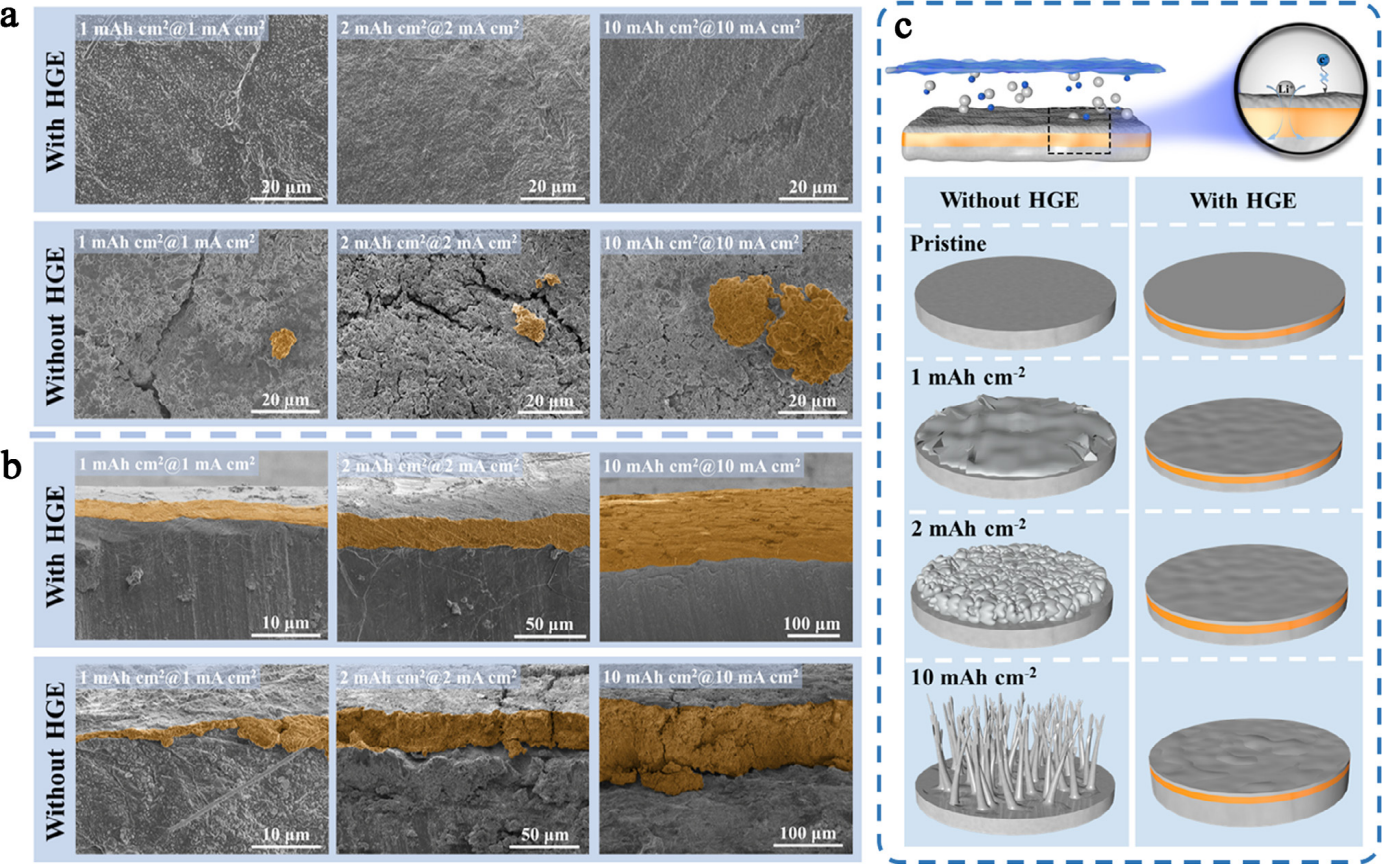
**Morphology of cycled LMAs with and without HGE protection.** The electrodes were first cycled with capacities of 1, 2, 10 mAh/cm^2^ at current densities of 1, 2, and 10 mA/cm^2^ for 50 cycles, respectively. The SEM images from the surface (a) and cross-section (b). (c) Schematic showing the proposed deposition mechanism enabling stable cycling of high current density with HGE.

**Fig. 5:**
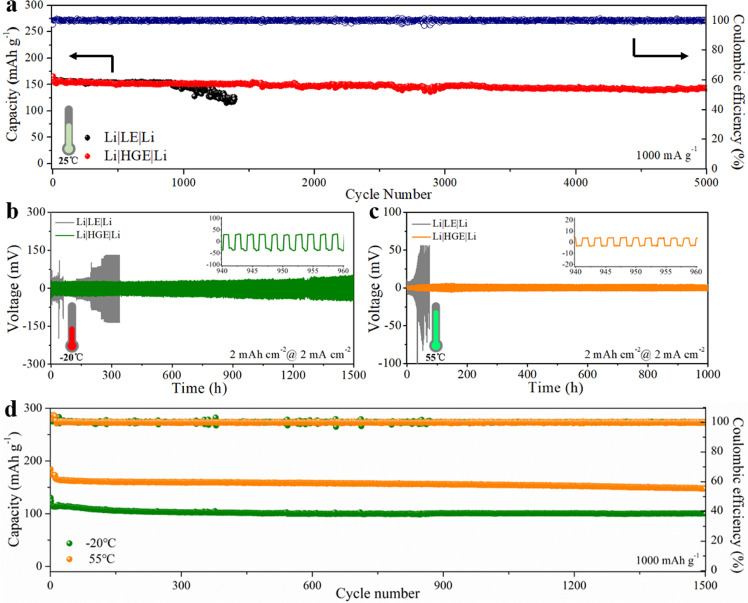
**Electrochemical performance of the full cells with LE and HGE at varying temperatures.** (a) Cycling performance of full cells coupled with LTO at room temperatures. The performance of Li|Li symmetric cells at (b) -20 °C and (c) 55 °C. The capacity was controlled to be 2 mAh/cm^2^ at a current density of 2 mA/cm^2^. (d) The cycle performance of Li|HGE|LTO cells at -20 °C and 55 °C.

The morphology and elemental mapping of HGE peeled off from the LMAs were characterized using high-angle annular dark-field (HAADF) imaging of aberration-corrected scanning transmission electron microscope (STEM). Fig. S11 indicates an amorphous structure of HGE with uniformly distributed Ga, In, Cl, and O elements, suggesting the uniform distribution of Li_x_Ga_86_In_14_ and LiCl in the polymer matrix. The selected area electron diffraction (SAED) in Fig. S12 confirms the presence of alloy and LiCl with low crystallinity. Fig. 2f displays the photos of the electrolyte gelation process with visualized polymerized electrolytes formation. The ionic conductivity decreased during polymerization and reached 3.6 × 10^−4^ S/cm after several minutes (Fig. S13), suggesting the fast polymerization of THF.

The performance of HGE was evaluated by assembling symmetric cells with LMA. Fig. 3a shows the stable cycling performance with the gel electrolyte over 1200 h at a high current density of 10 mA/cm^2^ and high capacity of 10 mAh/cm^2^, revealing a voltage hysteresis of ∼100 mV. However, the hysteresis with the liquid electrolyte rapidly increased after 200 h due to the Li dendrites growth and the accumulation of “dead Li”. The long cycling performance at various current densities is shown in Fig. S14. There is no apparent increase in voltage hysteresis after a 3000 h cycling of the cell with HGE, which is much better than that of the cells with the liquid electrolyte (less than 300 h). The selected magnified voltage profiles at different cycles are shown in the inset of Figs. 3**a, b** and S14, and well-defined voltage profiles with no “mass transport effect” caused by the stack of “dead Li” were also observed [41].

The rate capability of the symmetric cell with HGE under the varied current densities of 0.5, 1.0, 2.0, 4.0, and 10 mA/cm^2^ with a fixed capacity of 1 mAh/cm^2^ is shown in Fig. 3b. Although the overpotential gradually increased, stable voltage profiles and small overpotentials of ∼8, 10, 30, 50, and 90 mV were shown under the above current densities, showing outstanding rate performance. When the current density returned to 0.5 mA/cm^2^, the voltage profiles were resumed, which proved the superior stability. This should be ascribed to the hybrid layer on LMA surface effectively suppressed the growth of Li dendrites. The Li_x_Ga_86_In_14_ alloy is kinetically advantageous for Li nucleation and deposition, thereby providing a fast electron-ion transport pathway. Furthermore, LiCl salt and the in-situ generated gel synergistically construct a flexible and robust artificial SEI to mitigate the side reactions and suppress the dendrite growth.

To further demonstrate the effects of inhibiting Li dendrite growth by HGE, the evolution of Li anode surface during Li stripping/plating was recorded using an optical microscope at a current density of 5 mA/cm^2^. Uniform Li plating on the HGE protected LMA was achieved and indicated by the smooth surface upon successive Li plating (Fig. 3c). In contrast, several bulges appeared on bare Li after 20 minutes, suggesting an inhomogeneous Li deposition. The bare Li surface was covered with noticeable dendritic protrusions after 40 minutes and entirely covered after 60 minutes, while the HGE protected LMAs still exhibited flat and smooth surfaces with no dendrites.

The morphology of Li nucleation and growth was further studied by SEM, as shown in Fig. 4. The surface of the LMA with HGE is flat and smooth, even with the high deposition capacity of 10 mAh/cm^2^ and high current density of 10 mA/cm^2^, suggesting a uniform Li deposition (Figs. 4**a** and S15). However, a highly porous and rough morphology with large cracks and rugged Li deposition can be seen on the LMA cycled in a liquid electrolyte, which causes the increase of polarization, low Coulombic efficiency, and safety issues. With the magnified SEM images shown in Fig. S15, much more dendrites can be clearly observed. Fig. 4b displays the cross-sectional images of the cycled Li anode. A compact Li deposition layer is found with the modification of HGE, but the Li deposit exhibits a thick but highly loose structure in liquid electrolytes. The functions of HGE are schematically shown in Fig. 4c. In a liquid electrolyte, a higher Li diffusion kinetics and sluggish deposition rate lead to an accumulation of Li-ion at the surface, formation of deposition “hot spot” and thus the nonuniform deposition and dendrite growth. In contrast, the Li diffusion and transportation rates within the as-formed gel electrolyte are reduced considerably, and at the same time, the Li_x_Ga_86_In_14_ alloy kinetically promotes the Li nucleation and deposition by providing a fast electron and ion transport pathway [42]. Besides, the LiCl salt with an electronically insulating property helps construct a potential gradient in the HGE, which could promote Li^+^ migration through the gel electrolyte. Moreover, the organic gel network in HGE can also strengthen the mechanical properties, which can tolerate the volume changes during deposition to ensure integrity. These factors ensure uniform and dense Li deposition.

The prototype cell that consisted of Li metal, HGE and Li_4_Ti_5_O_12_ (denoted Li|HGE|LTO cell) was assembled and investigated. As shown in Fig. 5a, the capacity of the cell with the liquid electrode (denoted Li|LE|LTO cell) experiences a performance failure after 1000 cycles, which should be ascribed to the side reactions between the Li dendrites and electrolyte, consuming the Li-ions and electrolyte. In contrast, the Li|HGE|LTO cell exhibited excellent cyclability with a reversible capacity of 143.2 mAh/g over 5000 cycles (∼90% capacity retention, fading rate of 0.0021% per cycle). This should be derived from the stabilized interface and effectively suppress dendrite growth. The selected voltage profile cycles are shown in Fig. S16. The capacity loss from 100 to 5000 cycles was negligible, and the discharge-charge voltage gaps only increased slightly during the 5000 cycles.

The electrochemical performance of symmetric and Li|HGE|LTO cells over a wide temperature range (-20 to 55 °C) was investigated. It is noteworthy that the cells were directly subjected to electrochemical measurements at the set temperature without initial activation at room temperature. Fig. 5b shows that the voltage hysteresis of the symmetric cell with HGE is only 80 mV at -20 °C and a stable cycling more than 1500 h. However, cells with normal liquid electrolytes exhibited higher hysteresis of 130 mV initially and increased rapidly owing to the uneven Li deposition at low temperatures. Furthermore, the battery failure was observed at 55 °C only after a few cycles due to the high volatilization and poor thermal stability of the liquid electrolyte (Figs. S17**,**S18). The galvanostatic discharge–charge tests of Li|HGE|LTO cells were also performed at -20 and 55 °C and the results are shown in Fig. 5d. Except for the capacity fading at the several initial cycles, the cells with HGE showed high capacity retention of 93.5% at 55 °C and 88.8% at -20 °C after 1500 cycles under the high current density of 1000 mA/g, proving the improvement of electrochemical performance using this HGE over a wide temperature range. This should be ascribed to the lithiophilic alloy and LiCl layer which optimized the solvation structure and transportation–desolvation routes of Li-ions across the electrolyte, therefore suppressing the formation of Li dendrites, especially at low temperatures. In addition, the polymerized THF as an electrolyte exhibits low volatility, thus endowing stable performance at high temperatures.

## 4 Conclusion

In summary, a hierarchically structured polymerized electrolyte (HGE) was proposed to stabilize the LMBs over a wide temperature range. The HGE was prepared by simultaneously occurring polymerization-replacement-alloying reactions within the assembled cells, forming a hybrid layer of Li_x_Ga_86_In_14_, LiCl and polymerized THF attached to the LMA surface and the bulk polymerized THF gel electrolyte. The use of THF with low polarity and the hybrid layer with the ionic conductive Li_x_Ga_86_In_14_ alloy and LiCl salt ensure high ionic conductivity, especially under low temperatures. In addition, the polymerization of THF reduces the volatilization at higher temperatures and reinforces the mechanical strength of the HGE on LMA, guaranteeing the integrity of the hybrid layer and stable long-cycling performance. Thus, the HGE significantly decreases the polarization and extends the cycle life of the assembled symmetric and Li|HGE|LTO cells at a temperature range of -20 to 55 °C. The symmetric cells deliver a small overpotential of 100 mV at a high current density of 10 mA/cm^2^ with a high capacity of 10 mAh/cm^2^ for a more than 1200 h cycling. Besides, the low overpotentials of 12 mV at 55 °C and 80 mV at -20 °C at 2 mA/cm^2^ were also obtained after a 1000 h cycling. The Li|HGE|LTO showed stable cycling performance over 5000 cycles at room temperature and revealed the high capacity retention of 93.5% at 55 °C and 88.8% at -20 °C after 1000 cycles under the current density of 1000 mA/g. Overall, the HGE shows a promising solution to the gel electrolyte workable under a wide temperature range, which can be extended to other advanced battery systems such as Li-air, Li–S batteries, and other batteries with metal anodes, such as Na, K, Mg, and Zn.

## Declaration of Competing Interest

The authors declare that they have no conflicts of interest in this work.

## Appendix Supplementary materials

Image, application 1
