# Supplementary material for: Wide-temperature rechargeable Li metal batteries enabled by an in-situ fabricated composite gel electrolyte with a hierarchical structure
Source: Fundam Res. 2021 Nov 3;2(4):611–8. doi: 10.1016/j.fmre.2021.10.003 (PMC11197593; doi:10.1016/j.fmre.2021.10.003)
Supplement: Supplementary file 1 [file mmc1.docx]

**Supplementary Materials**

**Wide-temperature rechargeable Li metal batteries enabled by an in-situ fabricated composite gel electrolyte with a hierarchical structure**

Chao Ma ^a^, Xizheng Liu ^a,^*, Hui Geng ^a^, Xiaoshu Qu ^c^, Wei Lv ^b,^* and Yi Ding ^a^

^a^ Tianjin Key Laboratory of Advanced Functional Porous Materials, Institute for New Energy Materials and Low-Carbon Technologies, School of Materials Science and Engineering, Tianjin University of Technology, Tianjin 300384, China

^b^ Shenzhen Geim Graphene Center, Engineering Laboratory for Functionalized Carbon Materials, Tsinghua Shenzhen International Graduate School, Tsinghua University, Shenzhen, 518055, China.

^c^ Jilin Institute of Chemical Technology, Jilin 132073, China.

**Corresponding authors:**

E-mail: xzliu@tjut.edu.cn; lv.wei@sz.tsinghua.edu.cn


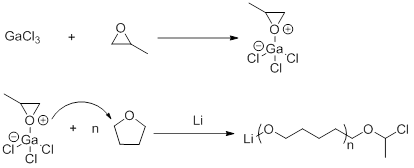


**Fig. S1.** The possible polymerization mechanism with PO.


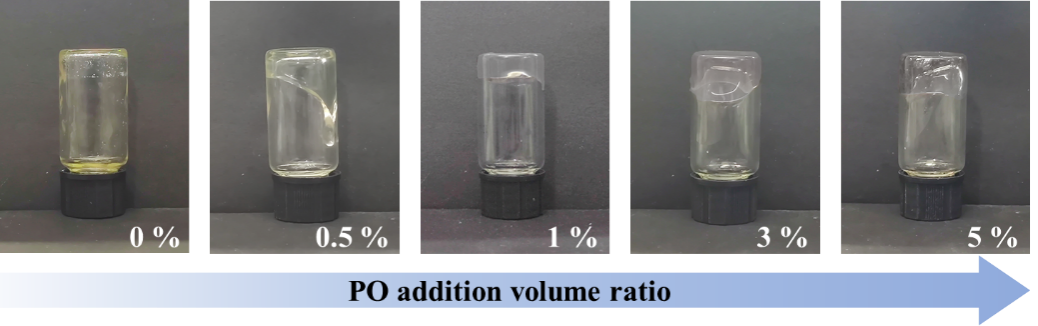


**Fig. S2.** Digital photos of THF polymerization with different PO content.

PO acts as a promoter during the polymerization reaction and its amount has been optimized. **Fig. S2** shows the digital photos of THF polymerization with different amounts of PO. It can be observed that the polymerization process becomes difficult with the decrease of PO amount. However, 1 vol% PO was enough to lead to complete polymerization, and thus, we performed the following experiments based on this result.


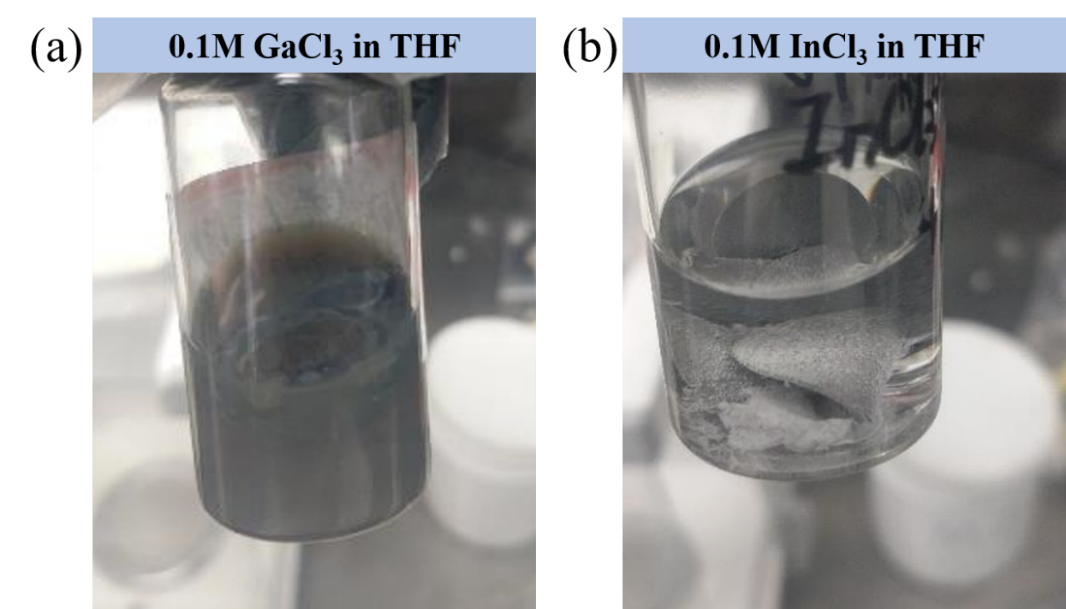


**Fig. S3.** Digital photos of Li foils after immersed in pure GaCl_3_(a) and InCl_3_(b) THF solutions for 12h.


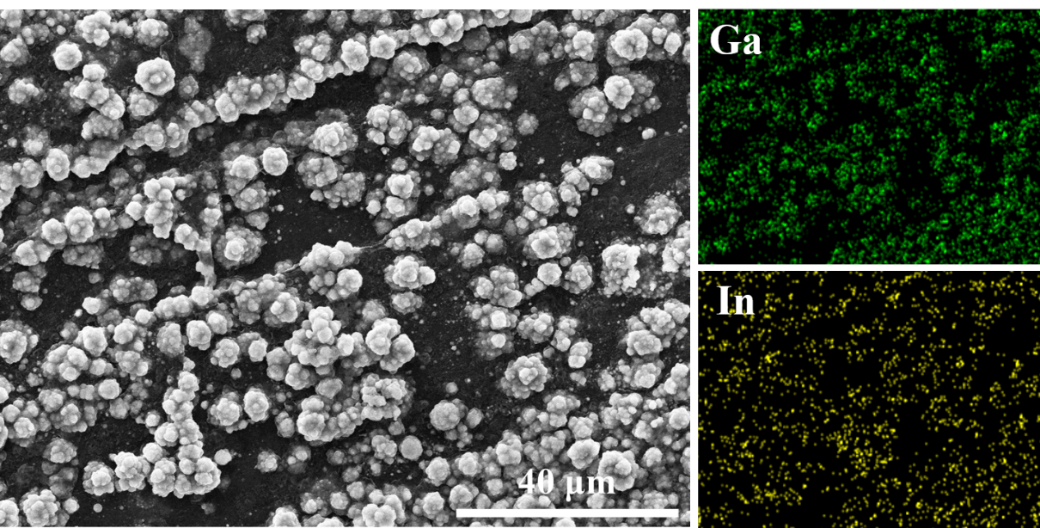


**Fig. S4.** SEM image and EDS mapping of Li foil treated with GaCl_3_/InCl_3_+DME.


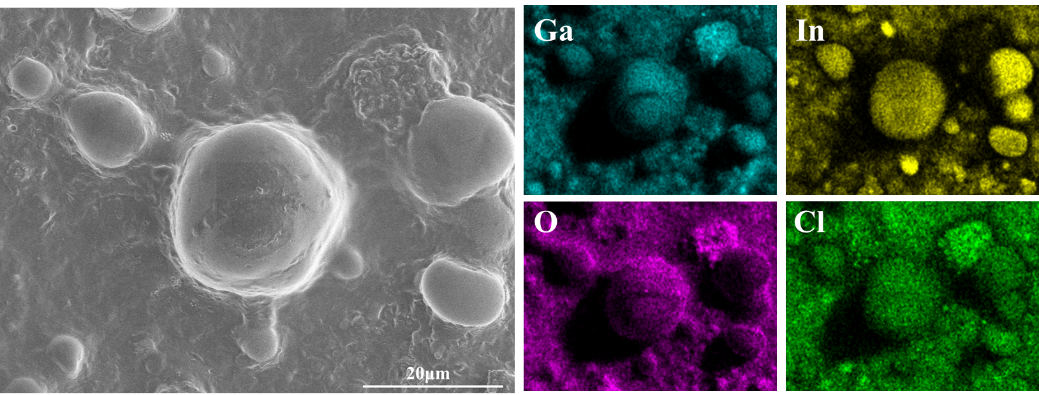


**Fig. S5.** SEM image and EDS mappings on the surface of HGE modified LMAs.


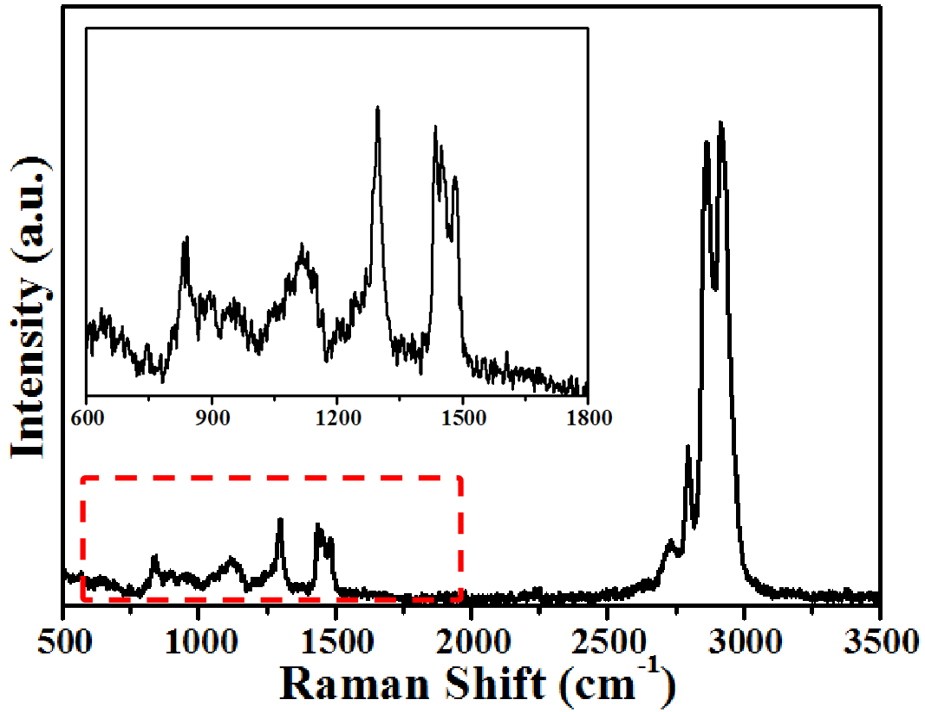


**Fig. S6.** Raman spectrum of HGEs.


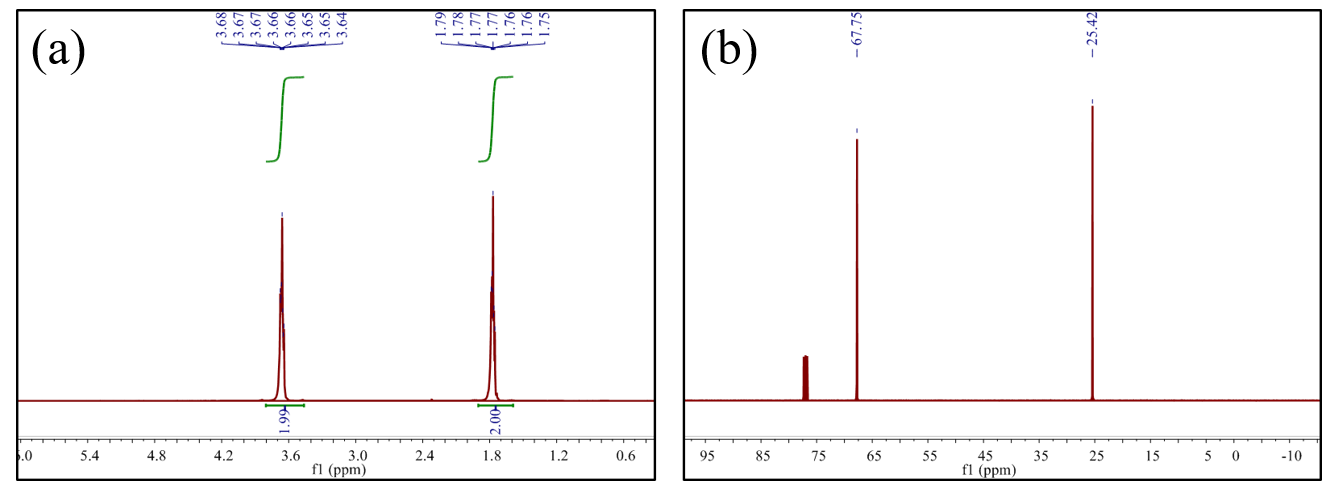


**Fig. S7.** The ^1^H NMR and ^13^C NMR spectra of pure THF.


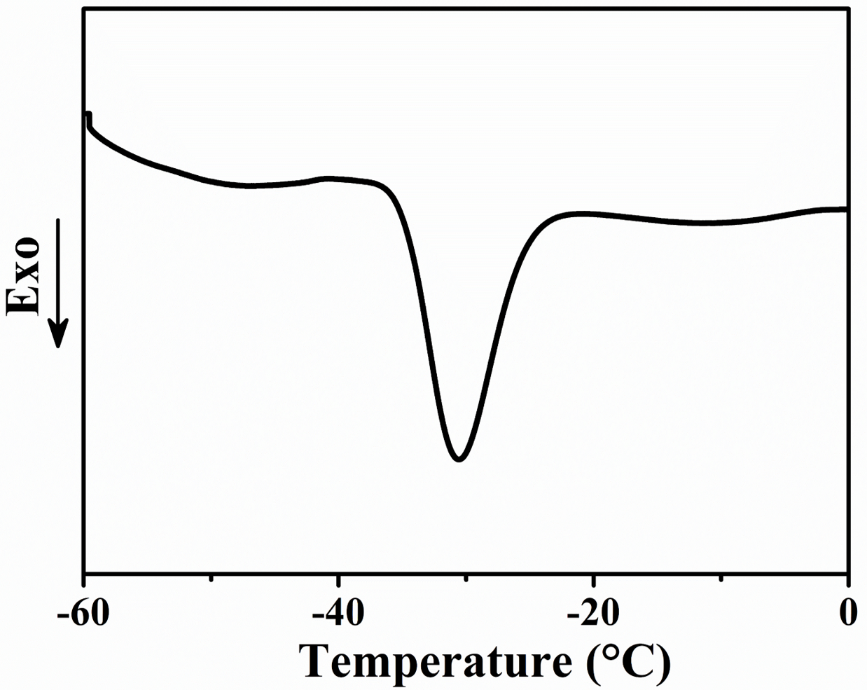


**Fig. S8.** DSC profile of HGEs.


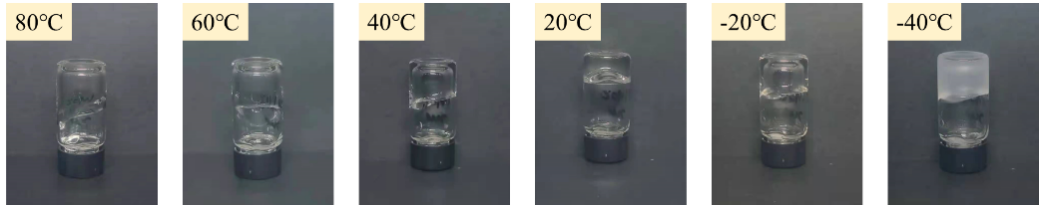


**Fig. S9.** Digital photos of HGEs at different temperatures.


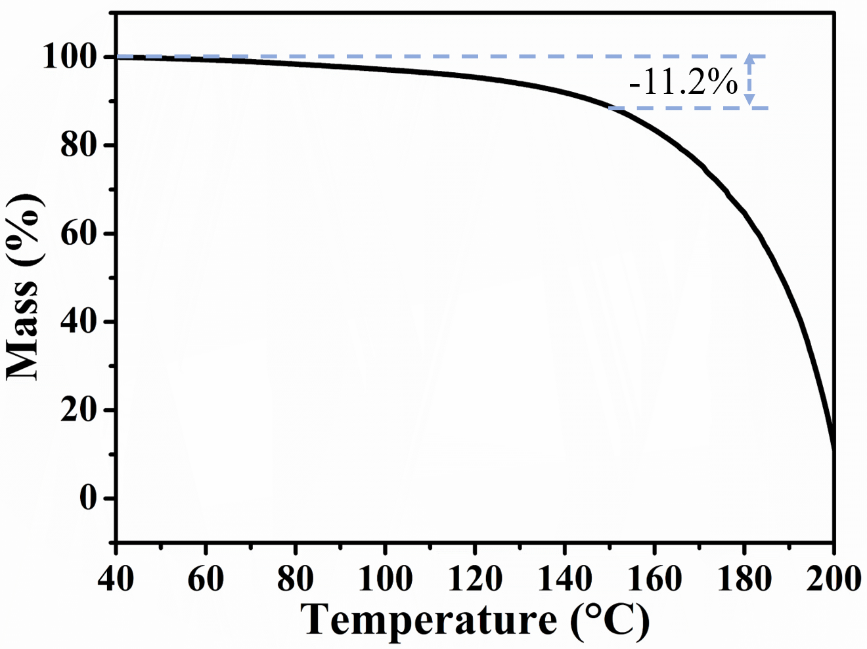


**Fig. S10.** Temperature-dependent weight loss of prepared HGE under N_2_ from 40 °C to 200 °C with a heating rate of 10 °C min^-1^.


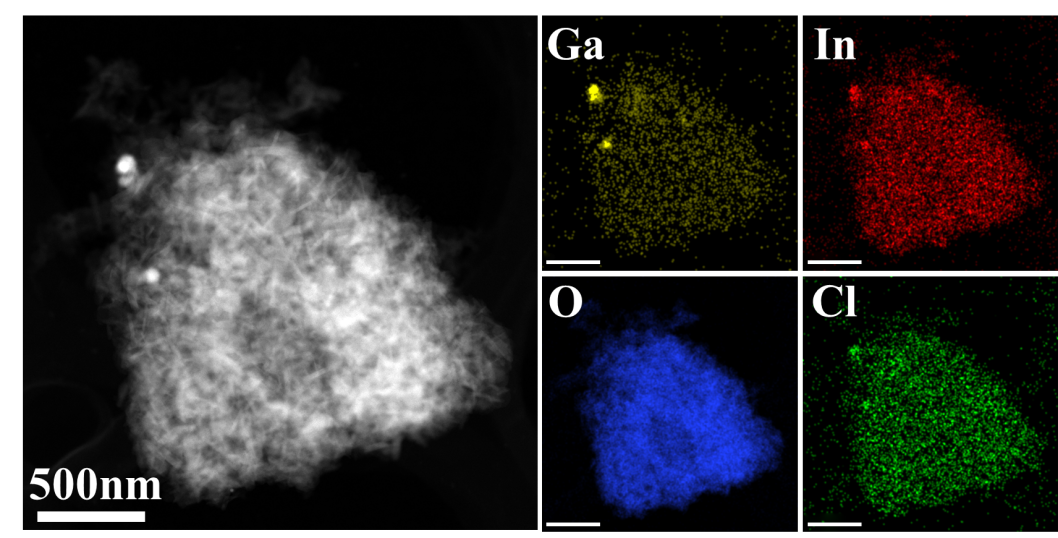


**Fig. S11.** HAADF-STEM images showing the morphology of HGE and the respective elemental mappings over the whole region. Uniform distributions of Li_x_Ga_86_In_14_ and LiCl can be observed.


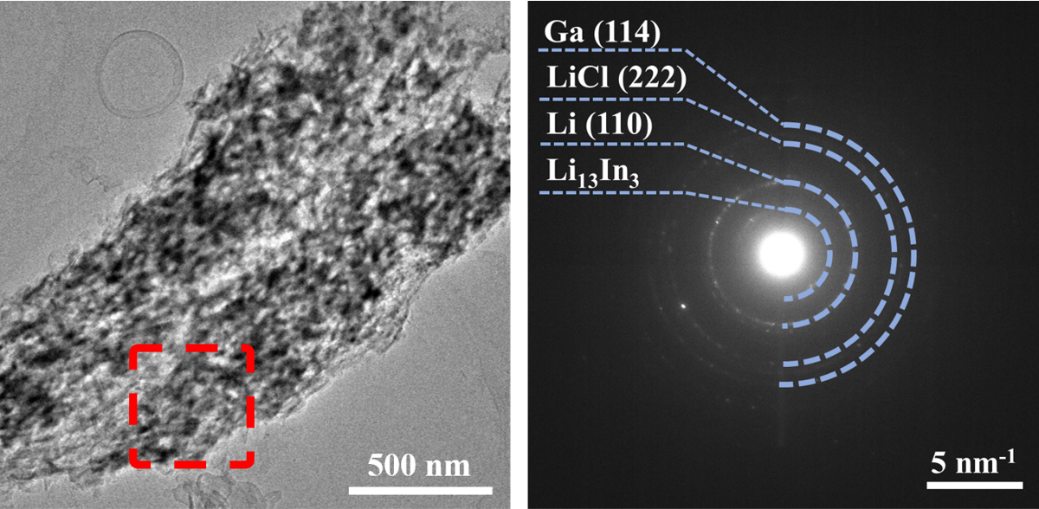


**Fig. S12.** HRTEM image and SAED of HGE.


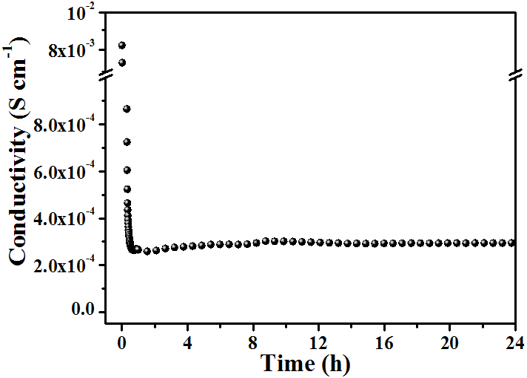


**Fig. S13.** The evolution of ionic conductivity during polymerization.


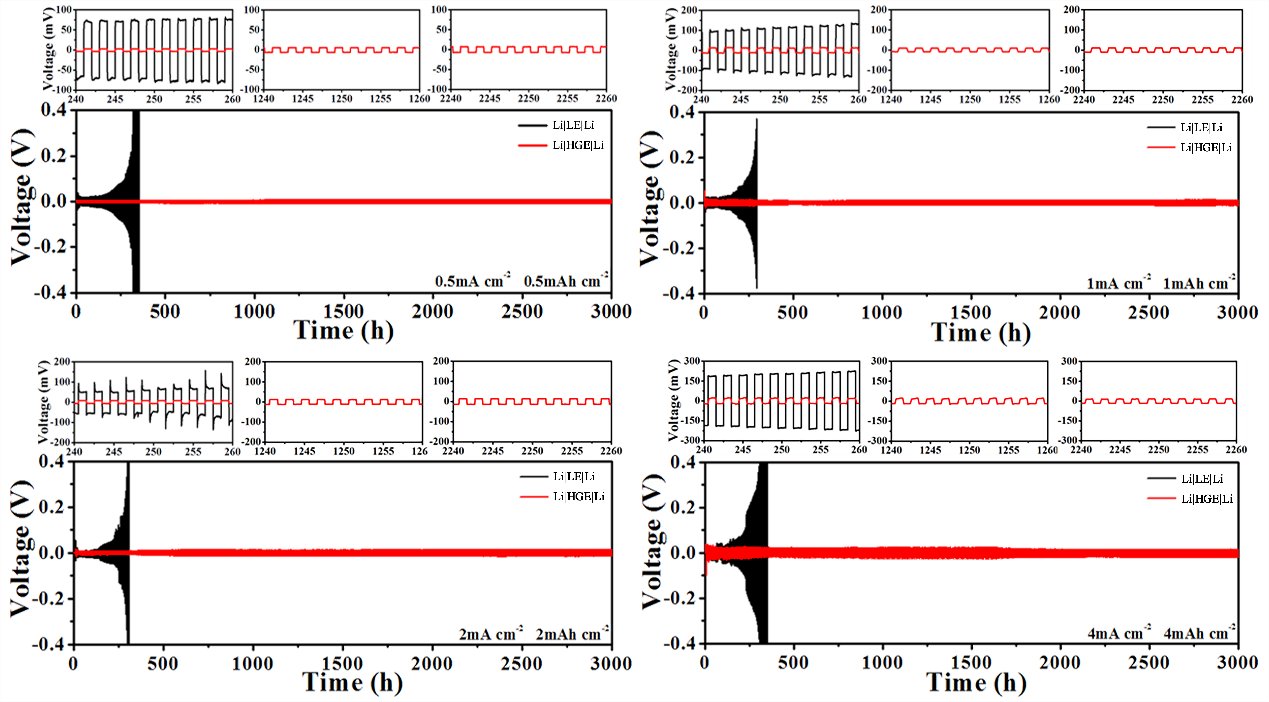


**Fig. S14.** Galvanostatic cycling performance of symmetric cells with LE or HGE at current densities of 0.5, 1, 2 and 4 mA/cm^2^ with limited stripping/plating time of 1h. The selected stripping and plating profiles at different states are above the individual figures.


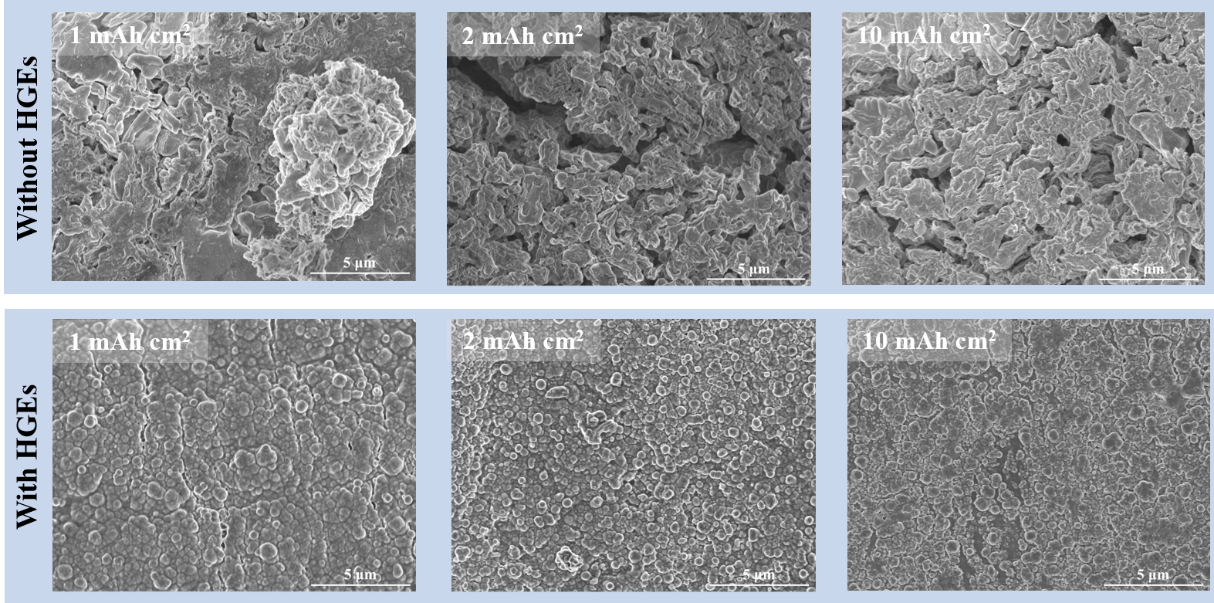


**Fig. S15.** The surface morphology after repeated cycles at different capacities.


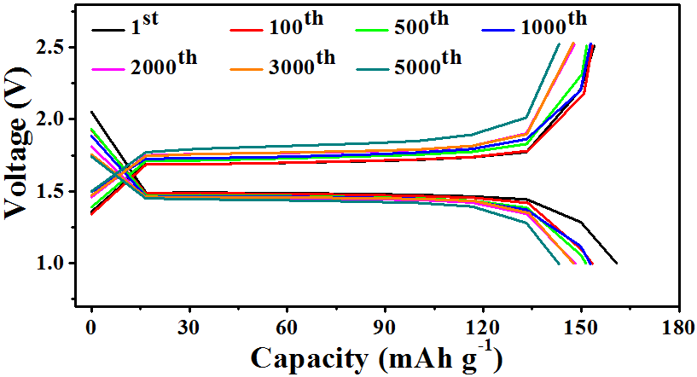


**Fig. S16.** Selected cycles of voltage profiles of Li|HGE|LTO cells at a current density of 1000 mAh/g.


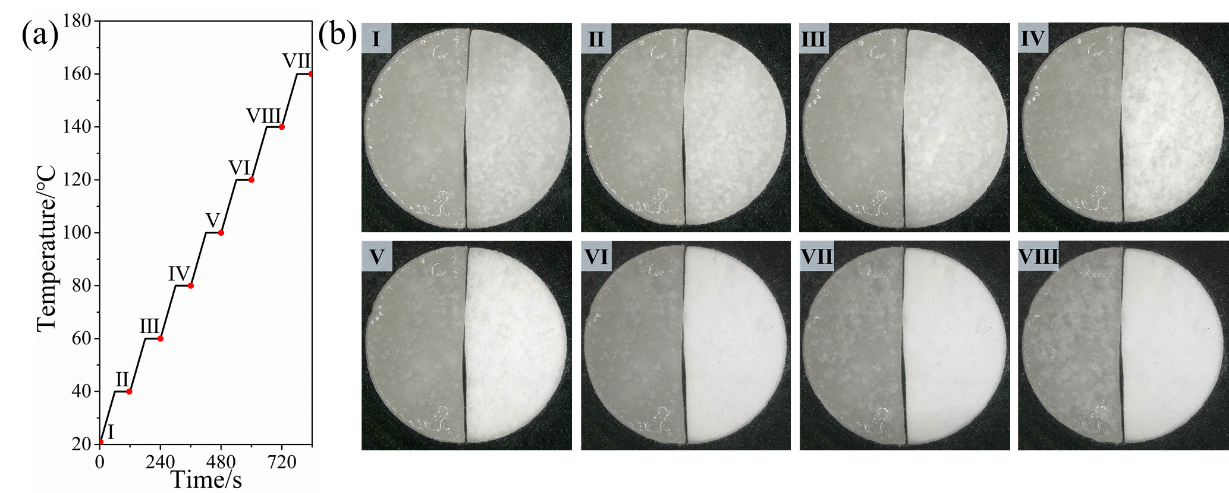


**Fig. S17.** The temperature dependence of different electrolytes. (a) Profile of the heating process. (b) The digital photos of electrolytes in the gel (left) and liquid (right) states under different temperatures.

In order to visualize the influence of temperature on the solvent of electrolyte, one piece of Glass-fiber was cut in half and infiltrated with HGEs and LEs, respectively. The evolution of the separators was recorded using an optical microscope equipped with an in-situ heating accessory. The temperature was controlled from 20 °C to 160 °C with a heating rate of 20 °C min^-1^. As shown in **Fig. S16**, HGE remained stable during heating with almost no solvent loss before 140℃. The phenomenon was consistent with the TGA result and indicated the good stability of prepared HGE at high temperatures. In contrast, LEs evaporated quickly and disappeared completely at 100℃. The instability of electrolytes will lead to degradation of electrochemical performance. In addition, the gas generated by the volatilization of electrolytes within the battery would lead to safety hazards. Thus, the in-situ solidification strategy of the electrolyte is crucial for improving the thermal stability and safety of batteries.


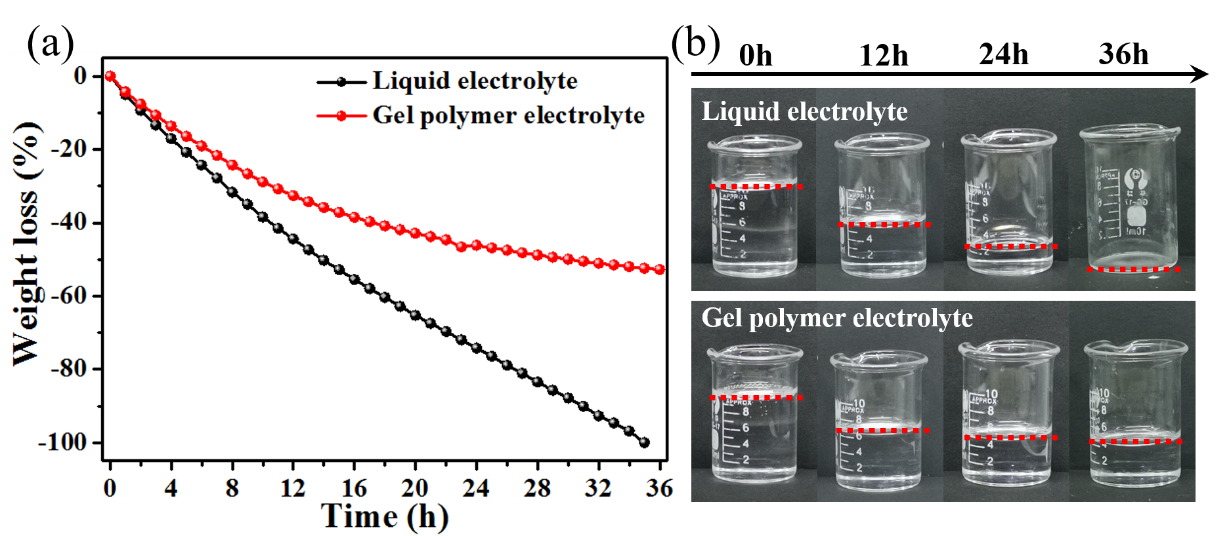


**Fig. S18.** (a) The comparison of weight losses of liquid electrolyte and polymerized electrolyte at ambient condition (about 20 °C). (b) The digital photos of electrolyte volatilization at liquid and gel states.
